# Entropy Contribution to the Line Tension: Insights from Polymer Physics, Water String Theory, and the Three-Phase Tension

**DOI:** 10.3390/e20090712

**Published:** 2018-09-16

**Authors:** Edward Bormashenko

**Affiliations:** Engineering Faculty, Chemical Engineering, Biotechnology and Materials Department, Ariel University, P.O. BOX 3, Ariel 407000, Israel; edward@ariel.ac.il

**Keywords:** line tension, entropic contribution, entropic force, orientation effect, hydrophobic substrate

## Abstract

The notion of three-phase (line) tension remains one of the most disputable notions in surface science. A very broad range of its values has been reported. Experts even do not agree on the sign of line tension. The polymer-chain-like model of three-phase (triple) line enables rough estimation of entropic input into the value of line tension, estimated as $\Gamma_{en}≅\frac{k_{B}T}{d_{m}}≅10^{-11}N$, where $d_{m}$ is the diameter of the liquid molecule. The introduction of the polymer-chain-like model of the triple line is justified by the “water string” model of the liquid state, predicting strong orientation effects for liquid molecules located near hydrophobic moieties. The estimated value of the entropic input into the line tension is close to experimental findings, reported by various groups, and seems to be relevant for the understanding of elastic properties of biological membranes.

## 1. Introduction

Surface tension is due to the special energy state of the molecules at a solid or liquid surface [1,2,3,4]. Molecules located at the triple (three-phase) line where solid, liquid, and gaseous phases meet are also in an unusual energy state [1,2,3,4]. The notion of line (three-phase) tension has been introduced by Gibbs. Gibbs stated: “These (triple) lines might be treated in a manner entirely analogous to that in which we have treated surfaces of discontinuity. We might recognize linear densities of energy, of entropy, and of several substances which occur about the line, also a certain linear tension” [5]. Notice that Gibbs emphasized the role of the entropy density, which will play the main role in our approach to the problem of line tension.

Even though the concept of line tension is intuitively clear, it remains one of the most obscure and disputable notions in surface science [6,7,8]. Researchers disagree not only on the value of line tension, but even on its sign. Experimental values of line tension Γ in the range of 10^−5^–10^−12^ N were reported [6,7,8,9,10,11]. Very few methods allowing experimental measurement of line tension were developed [9,10,11,12,13,14]. Marmur estimated a line tension as $\Gamma ≅4d_{m}\sqrt{\gamma_{SA}\gamma }\cot\theta_{Y}$, where *d_m_* is the molecular dimension, $\gamma_{SA},\gamma$ are surface energies of solid and liquid correspondingly, and *θ_Y_* is the Young angle. Marmur concluded that the magnitude of the line tension is less than 5 × 10^−9^ N, and that it is positive for acute and negative for obtuse Young angles [15,16]. However, researchers reported negative values of line tension for hydrophilic surfaces [14]. As for the magnitude of line tension, the values in the range 10^−9^–10^−12^ look realistic. Large values of Γ reported in the literature are most likely due to contaminations of the solid surfaces [3].

Let us estimate the characteristic length scale *l* at which the effect of line tension becomes important by equating surface and “line” energies: l≅Γ/γ=1−100nm. The effects relating to line tension can be important for nano-scaled droplets or for nano-scaled rough surfaces. However, these effects also may be important for the design of microfluidics circuits [6] and stabilization of the Cassie air-trapping wetting regime, enabling manufacturing of superhydrophobic and superoleophobic surfaces [17]. It was also shown that at millimeter-length scale, the gravitational potential provides a gravitational contribution Γ ≈ 1–10 µN, which is always positive [10]. The notion of line tension remains highly controversial, at least due to the conceptual difficulties, which arise because the interfaces between two phases are always diffuse and never sharp [18]. In the present article we try to estimate the role of the entropy contribution in constituting line (three-phase) tension.

## 2. “Water String Theory”, Insights from Polymer Physics and Entropy Contribution into Line Tension

It should be mentioned that calculation of line tension from first principles by MD and DFT (density functional theory) simulations is not a trivial task [19,20]. Nosonovsky in his recent article argued that both energy and entropy contribution should be taken into account for predicting surface and line tensions [21]. However, the entropy input remains “unseparated” within the reported MD and DFT calculations of the three-phase tension [19,20]. We will try to perform a rough estimation of input of the entropy factors in the entire value of the line tension. We restrict our treatment by case, when a sessile drop sits on hydrophobic (say polymer) surface, and the three-phase tension at the solid/liquid/vapor boundary appears. The line tension is also inherent for so-called liquid lens, arising at the liquid/liquid/vapor boundary [12,18] and giant biological vesicles, where a lipid bilayer membrane in its liquid state has the properties of a two-dimensional liquid [8].

**Figure 1:**
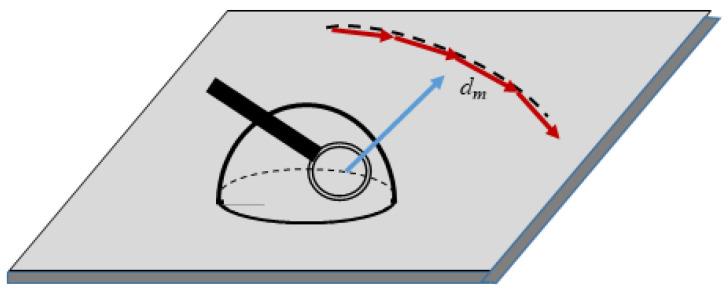
Three-phase line of the sessile droplet is approximated by the polymer chain with a diameter of the monomer *d_m_*, where *d_m_* is the diameter of the liquid molecule.

Let us start from the unobvious assumption that the line tension Γ may be split into “interactional” (denoted $\Gamma_{int}$) and entropy-inspired (denoted $\Gamma_{en}$) contributions:

(1) $$\Gamma =\Gamma_{int}+\Gamma_{en}$$

where $\Gamma_{int}$ is due to the interaction of molecules located at the triple line with surrounding ones, and $\Gamma_{en}$ is the entropy input into the three-phase tension. To estimate $\Gamma_{en}$, assume that molecules constituting the triple line form the quasi-polymer chain, as depicted in Figure 1. This idea originates from the “water string theory” presented and discussed in References [22,23,24]. X-ray absorption spectroscopy and X-ray Raman scattering demonstrated that water consists of structures with two strong H-bonds, one donating and one accepting, thus promoting formation of chain-like structures [22,23,24]. There is much theoretical and experimental evidence that water molecules are strongly oriented near hydrophobic moieties [25,26,27], and this is the case in our treatment (recall, that we restricted our consideration by sessile droplets, placed on hydrophobic surfaces). The authors of Reference [27] exploited the intensity vibrational sum-frequency generation (VSFG) technique pointing to an enhanced ordering of the water molecules surrounding the hydrophobic groups; in particular, the orientation was demonstrated for water molecules at the water/polydimethylsiloxane interfaces, thus justifying the chain-like model of the triple line, shown in Figure 1.

The use of polymer-chain model for the approximation of the triple enables immediate estimation of the entropy contribution into the line tension, according to Equation (2) (see Appendix A and Reference [28] for details).

(2) $$\Gamma_{en}≅\frac{k_{B}T}{d_{m}}$$

where $\Gamma_{en}$ is the entropy force necessary for stretching the pseudo-polymer chain built of liquid molecules, coinciding with the entropy input to the line tension, $k_{B}=1.38\times 10^{-23\;}\frac{J}{K}$ is the Boltzmann constant, *T* is the temperature and $d_{m}$ is the diameter of the molecule, representing the characteristic dimension of the “monomer” of the “polymer chain” [28]. Assuming for the water molecule $d_{m}≅2.75\times 10^{-10}m$ we derive from Equation (2) for water at ambient conditions $\Gamma_{en}≅1.5\times 10^{-11}N.$ The calculated value of the entropy-inspired line tension $\Gamma_{en}$ is comparable with the experimentally reported values of line tensions reported in References [6,7,9,10,11,19]. Thus, the input of entropy factors may be at least not negligible in constituting the value of three-phase tension.

The value of $\Gamma_{en}$ given by Equation (2) supplies the upper estimation for the entropic contribution to the line tension, because it is based on the “ideal chain approximation”, where the monomers do not interact [28]. For real chains, the interaction between monomers is not negligible and the estimation of the stretching force is supplied by Equation (3):

(3) $$\Gamma_{en}≅\frac{k_{B}T}{\xi }$$

where $\xi >d_{m}$ is the correlation length [28]. It is noteworthy, that the impact of the entropic term into the line tension is necessarily positive (the sign of the line tension remains debatable [5,6,7,11]). It is also recognized that the value of $\Gamma_{en}$ remains considerable for $\xi ≅5÷10\;d_{m}$ (values of $\Gamma ≅10^{-12}\;N)$ have been reported [9]. This means that the entropic input into the line tension may be important even when the interaction between molecules forming the three-phase line is considered. Equation (2) also predicts the decrease of the line tension with growth of the size of a liquid molecule *d_m_*. Regrettably, experimental data in the field are scarce, and do not enable clear conclusion on this item.

The rough estimation of the three-phase tension, given as Equations (2) and (3), is obvious from the dimensional considerations and it has been explicitly mentioned already in Reference [29]. The new aspect strengthened in the presented paper is identification of Equation (2) with the entropic contribution to the entire line tension seen as a sum of “interaction-” and “entropy”-inspired inputs. As with any other “entropic force”, the value of $\Gamma_{en}$ increases with temperature [21,28,30]. Experimental observations suggest the opposite temperature trend: both surface and line tensions are decreased with temperature [10,21,31]. This means that the “interaction” part of the line tension in the studied systems prevails on the “entropic” one. However, the input of entropy-inspired factors into the line tension may be not negligible, as has been suggested for membranes of biological vesicles [9]. At first glance, it seems that the phenomenon of line tension inherent for three-phase systems (liquid lens and sessile droplets) and twin-phase biological vesicles are quite different. However, closer inspection shows that an impact of the gaseous (vapor) phase on the line tension of liquid lenses and sessile droplets is minor. The proximity of the experimental values of the line tension ($\Gamma ≅10^{-10}÷10^{-11}\;N$) established for biological, vesicles, liquid lens and sessile drops is noteworthy [5,6,7,8]. Thus, it is reasonable to suggest that the polymer-chain model is applicable also to biological membranes built from phospholipid molecules containing two hydrophobic fatty acid "tails", promoting orientation effects [25,26,27].

## 3. Conclusions

The notion of line (three-phase) tension (which is important for constituting apparent contact angles [32,33], contact angle hysteresis [32,33,34,35] and stability of Cassie wetting states [17]) is revisited. It is suggested that the line tension is built from “interaction” and “entropic” contribution, which are usually unseparated under calculation of the three-phase tension. For the estimation of the entropic input into line tension the three-phase line is approximated by a polymer chain [28,29]. This model is justified by novel experimental data indicating strong orientation effects for liquid molecules located near hydrophobic moieties [22,23,24,25,26,27]. Thus, the polymer-chain model may be successful for sessile droplets placed on hydrophobic (say, polymer) solid substrates. If the polymer-chain model is adopted for the triple (three-phase) line the entropic contribution to the line tension is crudely estimated as $\Gamma_{en}≅\frac{k_{B}T}{\xi }$, where ξ is the correlation length [28]. If the triple line is seen as an ideal polymer chain, the entropic input is estimated as $\Gamma_{en}≅\frac{k_{B}T}{d_{m}}$, where $d_{m}$ is the diameter of the liquid molecule, representing the characteristic dimension of the “monomer” of the “polymer chain”. Simple estimations supply for the entropic input into the line tension the value of $\Gamma_{en}≅1.5\times 10^{-11}\;N$, which is comparable with the reported experimental data [6,7,9,10]. We conclude that the entropic contribution into the line tension is not negligible. It appears that the entropic contribution to line tension may be essential and even dominating in biological systems, such as giant lipid vesicles, where the decisive role of the entropy input in the elastic properties of biological membranes was demonstrated both experimentally [36,37] and theoretically [38]. Notice that in the case of biological membranes the line tension is not three-phase, but the liquid/liquid phases inspires the phenomenon. Evans and Rawicz stated in Reference [37] that “in recent years, it has become apparent that thin condensed-fluid membranes behave as 2D generalizations of linear flexible polymers” and demonstrated that the elastic properties of vesicles are entropy-dominated.

## Figures and Tables

**Figure A1:**
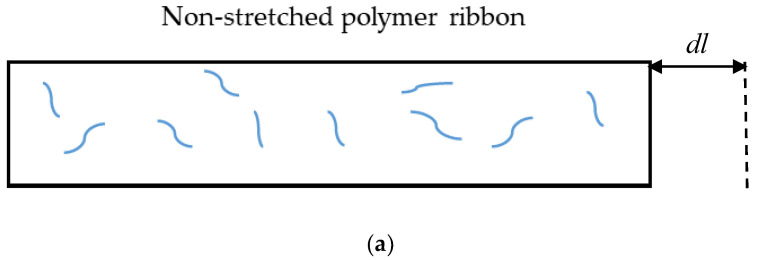

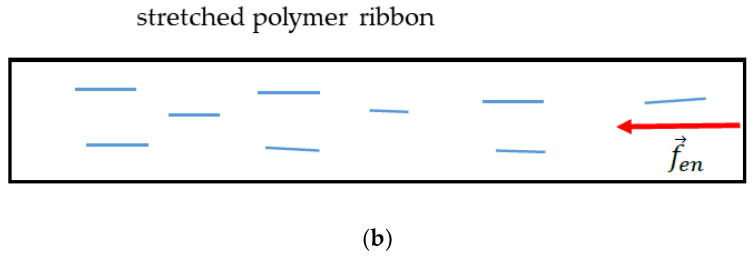
The origin of the entropic force ${\vec{f}}_{en}$ is exemplified with the stretching of the polymer ribbon. Sketch (**a**) depicts the non-stretched polymer ribbon. Molecules of polymer are non-stretched. Sketch (**b**) depicts the same ribbon when stretched (the stretch is *dl*). Molecules of polymer are stretched; the entropy of the ribbon S is correspondingly decreased; $\left|f_{en}\right|=-T\frac{\Delta S}{dl}>0$.

## Appendix A. Origin of the Entropic Elastic Forces

Consider isothermal stretching of the polymer ribbon, shown in Figure A1. The Helmholtz free energy of the ribbon is *F* = *U* − *TS* (*U* is the internal energy of the ribbon); thus, the change in the Helmholtz free energy under isothermal stretching equals [28,29]:

(A1) ΔF=−TΔS

Notice, that ΔS<0 takes place (the entropy of polymer chains is decreased under stretching [28,29]), hence ΔF>0. When the stretch equals *dl* the modulus of the entropic elastic force *f_en_* is given by Equation (A2):

(A2) $$\left|f_{en}\right|=-T\frac{\Delta S}{dl}>0$$

It is recognized from Equation (A2) that $\left|f_{en}\right|\sim T$ is true for entropic elastic forces.
